# Significance of clinical phenomes of patients with COVID‐19 infection: A learning from 3795 patients in 80 reports

**DOI:** 10.1002/ctm2.17

**Published:** 2020-04-04

**Authors:** Linlin Zhang, Diane C. Wang, Qihong Huang, Xiangdong Wang

**Affiliations:** ^1^ Zhongshan Hospital Institute of Clinical Science Fudan University Shanghai Medical School Shanghai China; ^2^ Department of Emergency Sunshine Coast University Hospital Birtinya Queensland Australia; ^3^ Jinshan Hospital Center for Tumor Diagnosis & Therapy Jinshan Hospital Fudan University Shanghai Medical School Shanghai China; ^4^ Shanghai Engineering Research Center of AI Technology for Cardiopulmonary Diseases Shanghai China; ^5^ Shanghai Institute of Clinical Bioinformatics Shanghai China

**Keywords:** acute lung injury, clinical phenome, COVID‐19, lung

## Abstract

A new coronavirus SARS‐CoV‐2 has caused outbreaks in multiple countries and the number of cases is rapidly increasing through human‐to‐human transmission. Clinical phenomes of patients with SARS‐CoV‐2 infection are critical in distinguishing it from other respiratory infections. The extent and characteristics of those phenomes varied depending on the severities of the infection, for example, beginning with fever or a mild cough, progressed with signs of pneumonia, and worsened with severe or even fatal respiratory difficulty in acute respiratory distress syndrome. We summarized clinical phenomes of 3795 patients with COVID‐19 based on 80 published reports from the onset of outbreak to March 2020 to emphasize the importance and specificity of those phenomes in diagnosis and treatment of infection, and evaluate the impact on medical services. The data show that the incidence of male patients was higher than that of females and the level of C‐reaction protein was increased as well as most patients' imaging included ground‐glass opacity. Clinical phenomes of SARS‐CoV‐2 infection were compared with those of SARS‐CoV and MERS‐CoV infections. There is an urgent need to develop an artificial intelligence‐based machine learning capacity to analyze and integrate radiomics‐ or imaging‐based, patient‐based, clinician‐based, and molecular measurements‐based data to fight the outbreak of COVID‐19 and enable more efficient responses to unknown infections in future.

The coronavirus (CoV) is a single‐stranded enveloped virus, belonging to the family Coronaviridae, with a positive‐sense RNA genome about 26‐32 kb in size. It is widespread among humans and other mammals, such as camels, bats, masked palm civets, mice, dogs, and cats.^1^, ^2^ Two viruses, severe acute respiratory syndrome coronavirus (SARS‐CoV)^3^ and MERS‐CoV,^4^ have had a significant impact on humans over the past two decades, although most CoVs cause mild clinical symptoms in humans. In December 2019, pneumonia with an unknown cause broke out in Wuhan, China,^5^ which was different from SARS‐CoV and MERS‐CoV. This new type of CoV is the seventh family member of CoV to infect humans.^6^ The International Virus Classification Commission has renamed the virus, previously temporarily named 2019‐nCoV, as severe acute respiratory syndrome coronavirus‐2 (SARS‐CoV‐2), and WHO has announced a new name for this epidemic: coronavirus disease (COVID‐19).^7^ Genome sequencing found it to be a beta CoV with at least 70% genetic‐sequence similarity to SARS‐CoV. We summarized clinical phenomes of 3795 patients with SARS‐CoV‐2 infection from 80 international and national reports (Supporting Information Table 1) since the outbreak, mainly including complaints and signs (3795 patients in Table 1), biochemical measurements (3085 patients in Table 2), and imaging information with an autopsy report (2856 patients in Tables 3 and 4). There is only one autopsy report because there is less information in this field, but understanding the pathogenesis of COVID‐19 may also help doctors make treatment plans, so we also included them.

**TABLE 1:** Patient complaint‐ and physical examination‐based phenomes

Phenomes	Sum/number	Phenomes	Sum/number	Phenomes	Sum/number	Phenomes	Sum/number	Phenomes	Number/mean ± SE
General information		Nasal congestion	82	Myalgia or arthralgia	365	Hepatitis B infection	26	Arrhythmia	23
Patient number	3795			Chill	188	COPD	51	ARDS	99
Male	2081	Little phlegm	57	Abdominal pain and diarrhea	7	Chronic lung disease	26	AKI	5
Female	1705	Sputum	586	Dyspnea	188	Cardiac or cardiovascular disease	205	Respiratory failure	11
Age	46.48	Blood in sputum	3	Chest tightness and dyspnea	83	Never smoked	927	Hepatic insufficiency	2
Clinical classification		Myalgia or fatigue	103	Shortness of breath	323	Former smoker	29	Renal insufficiency	14
Mild type	37	Mild headache and dizziness	8	Chest distress	136	Current cigarette smoker	196	Secondary infection	4
Moderate type	487	Coarse breath sounds	3	Chest pain	41	Cerebrovascular disease	32	DIC	1
Severe type	331	Rhinorrhea	65	Rhonchi	4	Cardiovascular and cerebrovascular	53	Rhabdomyolysis	2
Critically ill type	8			Moist rales	2	Malignancy	24	Co‐infection	
Signs and symptoms		Sneeze	5	Conjunctivitis	1	Chronic kidney disease	25	Other viruses	0
No obvious symptom	184	Weakness	7	Conjunctival congestion	9	HIV infection	2	Bacteria	1
Fever	1703	Sore throat	297			Bacterial co‐infections	2	Fungus	4
Higher temperature	86.8	Agrypnia	22	Hemoptysis	2	Digestive system disease	31		
Normal	2	Gastrointestinal symptoms		Confusion	9	Endocrine system disease	27	PaO_2_ (83‐108)	93.76 ± 4.40
<37.3°C	742	Loss of appetite	208	Upper airway congestion	8	Nervous system disease	2	PaO_2_:FIO_2_ (400‐500)	207.84 ± 35.92
37.3‐38.0°C	541	Nausea and vomit	216	Signs of infection		Immunodeficiency	6	SaO_2_ (≥95%)	75.43 ± 5.79
38.1‐39.0°C	483	Nausea	60	Throat congestion	19	Hyperlipidemia	22	Decreased	9
>39.0°C	132	Vomit	20	Tonsil swelling	23	Cholelithiasis	6	PaCO_2_ (35‐48)	36.69 ± 1.10
Fatigue	943	Diarrhea	187	Enlargement of lymph nodes	2	Thyroid diseases	9	Heart rate mean	91.85 ± 0.92
Headache	290	Gastrointestinal reaction	1	Rash	2	Urolithiasis	3	Respiratory mean	21.70 ± 0.15
Headache and mental disorder symptoms	13	Abdominal pain	14	Comorbidities		Stroke	3	MAP	90.00 ± 0.00
Dizziness	39	Belching	7	Diabetes	214	Complications		Systolic pressure	131.82 ± 1.36
Dizziness or headache	7	Constipation	48	Hypertension	415	Shock	32	Diastolic pressure	79.57 ± 0.94
Cough	1986	Hemoptysis	17	Chronic liver disease	40	Acute cardiac injury	16		

Abbreviations: AKI, acute kidney injury; ARDS, acute respiratory distress syndrome; COPD, chronic obstructive pulmonary disease; DIC, disseminated intravascular coagulation; MAP, mean arterial pressure; PaCO_2_, partial pressure of carbon dioxide in artery; PaO_2_:FIO_2_, partial pressure of oxygen:fraction of inspiration O_2_; PaO_2_, partial pressure of oxygen; SaO_2_, arterial oxygen saturation.

**TABLE 2:** Biochemical‐ and molecular measurements‐based phenomes

Phenomes	Number/mean ± SE	Phenomes	Number/mean ± SE	Phenomes	Number/mean ± SE	Phenomes	Number/mean ± SE	Phenomes	Number/mean ± SE
Inflammatory cells		Platelets (125‐350)	180.92 ± 1.94	Amyloid A (<10)	237.73 ± 55.51	D‐dimer (<0.55)	16.14 ± 4.15	Creatine kinase (<171)	103.01 ± 5.05
Patient number	3085	Increased	10	Increased	14	Increased	262	≥200 U/L	90
WBC (3.5‐9.5)	5.53 ± 0.05	Decreased	24	Normal	4	Normal	2	Creatine kinase–MB (<25)	13.03 ± 1.53
Increased	81	Normal	1	Ferritin (21.0–274.7)	808.7 ± 0.00	FDP (1‐5)	4.00 ± 0.00	CTnl (0‐0.1)	0.53 ± 0.22
Decreased	230	Haemoglobin (110‐160)	137.10 ± 1.04	CD3 (723‐2737)	363.74 ± 90.37	AT (80‐120)	91.00 ± 0.00	LDH (125‐243)	277.98 ± 6.12
Normal	202	Decreased	1	Decreased	28	Blood biochemistry		Increased	13
Neutrophils (1.8‐6.3)	15.37 ± 1.97	Normal	10	CD4 (404‐1612)	244.08 ± 34.04	Lactate (0.5–2.0)	1.20 ± 0.05	Myoglobin (0‐110)	58.69 ± 4.59
Increased	43	Eosinophils (0.02‐0.52)	0.05 ± 0.00	Decreased	60	Albumin (40‐55)	34.89 ± 0.94	Glucose (3.9‐6.1)	8.13 ± 0.49
Decreased	47	Increased	2	IL‐6 (0.1‐2.9)	149.66 ± 62.27	Alanine ATase (9‐50)	32.95 ± 0.97	BNP (0‐23.1)	43.90 ± 0
Normal	54	Decreased	14	CD8 (220‐1129)	247.08 ± 32.54	Increased	160	HT‐I (<26.2)	4.90 ± 0.75
Lymphocytes (1.1‐3.2)	1.72 ± 0.14	Normal	29	Decreased	20	Aspartate ATase (15‐40)	35.48 ± 0.78	K (3.5‐5.5)	4.05 ± 0.10
Increased	1	Infection‐related biomarkers		CD19	72.77 ± 15.89	Increased	171	Decreased	10
Decreased	347	Procalcitonin (<0.05)	0.25 ± 0.04	CD 16 (<56)	64.21 ± 20.61	TG (<1.70)	1.14 ± 0.00	Na (136‐146)	137.72 ± 0.35
Normal	24	Increased	71	Coagulation function		Total bilirubin (4.7‐24)	15.67 ± 1.72	Cl (96‐106)	98.77 ± 1.00
RBC (3.5‐5.5)	4.45 ± 0.14	Normal	95	Thrombin time s (16‐18)	28.90 ± 0.00	Increased	78	Decreased	15
Monocytes (0.1‐0.6)	0.46 ± 0.04	ESR (20 mm/h)	35.44 ± 1.21	APTT (25.1‐36.5)	32.02 ± 0.62	BUN (2.8‐7.6)	4.74 ± 0.00	PH (7.35‐7.45)	7.42 ± 0.00
Increased	10	CRP (0‐10)	38.01 ± 1.49	PT (9.4‐12.5)	12.58 ± 0.16	Serum urea (2.5‐7.1)	3.80 ± 0.00	Increased	21
Decreased	3	Increased	870	Fibrinogen (2‐4)	5.23 ± 0.52	Decreased	3	decreased	4
Normal	16	Normal	66	Increased	12	Creatinine (64‐104)	68.19 ± 1.45	Lactate (0.5‐1.6)	1.30 ± 0.00
						Increased	15		
						Decreased	2		

Abbreviations: APTT, activated partial thromboplastin time; AT, angiotensin; BNP, brain natriuretic peptide; BUN, blood urea nitrogen; CD16, cluster of differentiation 16; CD19, cluster of differentiation 19; CD3, cluster of differentiation 3; CD4, cluster of differentiation 4; CD8, cluster of differentiation 8; Cl, chlorine; CRP, C‐reactive protein; CTn1, cardiac troponin 1; ESR, erythrocyte sedimentation rate; FDP, fibrin degradation product; HT‐I, hypersensitive troponin I; IL‐6, interleukin‐6; K, potassium; LDH, lactate dehydrogenase; M, mean; Na, sodium; PH, potential of hydrogen; PT, prothrombin time; RBC, red blood cell count; TG, triglyceride; WBC, white blood cell count.

**TABLE 3:** Imaging‐based phenomes, eg radiographs, ultrasounds, pathology

Phenomes	Sum/number	Phenomes	Sum/number	Phenomes	Sum/number	Phenomes	Sum/number
Phenomes	SUM	Pleural impairment	2	Right	28	Crazy paving pattern	51
Patient number	2856	Pleural thicken	15	Left upper lobe	187	Peripheral distribution	7
Normal	75	Pleural effusion	21	Left lower lobe	285	Peribronchovascular	1
Patchy	491	Pericardial effusion	8	Left	19	Scattered opacities	1
GGO/consolidation	18	Lymphadenopathy	9	Upper lobes	43	Ill‐defined	1
GGO without consolidation	12	Lobe affected		Middle lobe	30	Other findings	
GGO with consolidation	113	0	24	Lower lobes	51	Discrete pulmonary nodules	14
Consolidation	119	1	102	Predominant distribution		Lymph node enlargement	9
Parenchymal abnormalities	3	2	59	Anterior	9	Fibrous stripes	35
Spider web sign	20	3	56	Posterior	42	Cystic changes	8
Ill‐defined margins	124	4	62	Inferior	2	Bronchiectasis	11
GGOs with interstitial and/or interlobular septal thickening	147	5	136	Peripheral	152	Adjacent pleura thickening	77
GGO	999	>2 lobes	70	Subpleural region	45	Interlobular septa thickening	1
Patchy/punctuated GGO	105	Unilateral lung	107	Central	5	Interstitial abnormalities	143
GGO were bilateral	182	Bilateral lung disease	1119	Diffuse distribution	43	Pleura thickening	1
GGO involving the posterior lungs	41	Bilateral upper lobes	67	Peribronchial distribution	3	Qualitative change on follow‐up chest CT	
GGO involving the peripheral	44	Bilateral lower lobes	72	Both central and peripheral	1	No change	1
Air bronchogram	165	Frequency of lobe involvement		Mixed distribution	26	Disease improvement	1
Bronchial wall thickening	9	Right upper lobe	181	Opacification distribution and patterns		Mild disease progression	7
reticulation	23	Right middle lobe	124	Rounded morphology	7	Moderate disease progression	2
		Right lower lobe	306	Linear opacities	58	Severe disease progression	1

Abbreviation: GGO, ground‐glass opacity.

**TABLE 4:** Pathological characteristics of postmortem biopsies

Phenomes	Sum/number	Phenomes	Sum/number
Patient number	1	Viral cytopathic‐like changes	1
Histological examination		Intranuclear or intracytoplasmic viral inclusions	0
Bilateral	1	Liver biopsy	
Alveolar damage	1	Microvesicular steatosis	
Cellular fibromyxoid exudates	1	Mild	
Desquamation of pneumocytes	1	Moderate	1
Hyaline membrane formation	1	Severe	
Pulmonary oedema	1	Lobular and portal activity	
Interstitial mononuclear inflammatory infiltrates	1	Mild	1
Multinucleated syncytial cells	1	Moderate	
Atypical enlarged pneumocytes	1	Severe	
Large nuclei	1	Interstitial mononuclear inflammatory infiltrates	1
Amphophilic granular cytoplasm	1	Heart tissue	
Nucleoli in the intra‐alveolar spaces	1	No substantial damage	1

The most common clinical phenomes of patients were fever, malaise, dry cough, shortness of breath, and respiratory distress, as reported initially.^8^ One study showed that about 66% of patients had a history of exposure to a wet market in Wuhan, of whom 73% were males and <50% suffered from underlying diseases, including diabetes, high blood pressure, and cardiovascular diseases. More than 90% had fever ranging between 38 and 39°C and bilateral chest abnormalities on radiography, 76% had cough, 63% had reduced lymphocyte number, 55% dyspnea, 44% fatigue, 28% sputum, 25% low white blood cell counts, as well as other clinical features, including headache, hemoptysis, diarrhea, or hypertension.^9^ It would be more beneficial to set up a digital evaluation score system to present the variety of clinical phenomes and integrate with molecular multi‐omics data. Table 1 shows that the number of male patients was higher than that of females, with an average age of 46.48 years. The most common phenomes are fever, cough, fatigue, sputum, myalgia, sore throat, and shortness of breath, as summarized from more than 80 published studies (Supporting Information Table 1).

In addition, clinical phenomes related with gastrointestinal phenomes, for example, nausea or vomiting were also noticed.^10^ The history of exposure to susceptibility factors is one of the critical factors in the diagnosis, including visit to the seafood market, travel through infected area, or contact with people with infection.^11^ Radiomics‐based phenomes become more important in the early diagnosis and dynamic monitor of disease progression. Imaging phenomes of SARS‐CoV‐2‐infected pneumonia can be detected about 9 days after the first visit to the clinic, and turbid basal striations in both lower lobes of lungs are shown at about 10 days, during which the rale lung sound could be heard.^10^ Multiple peripheral ground‐glass opacities appeared in both lobes of lungs in some patients.^12^ From the preliminary analysis of about 80 clinical reports (Supporting Information Table 1), about 75% (2856/3795 patients) had chest CTs that showed ground‐glass opacity, patchy shadowing, and bilateral lower‐lobe inflammation as common radiographic features of patients with COVID‐19 infection. The progression of COVID‐19‐infected lung injury is described by increased density, profusion, confluence, and pleural effusion in chest radiographs^6^ (Table 3). In a study of 159 asymptomatic patients, we collected chest CT imaging of most patients that showed ground‐glass opacity shadows, of which 82 were pure ground‐glass opacity shadows, and 17 cases presented ground‐glass opacity with consolidation. Among patients with asymptomatic infections, a chest CT scan is particularly important to facilitate early detection of suspected cases and guide early isolation. There is an urgent need to develop a mode of artificial intelligence analysis to generate more detailed imaging phenomes and digitalize the image abnormality of density, size, distribution, and geometric figures.

Table 2 demonstrates that the number of lymphocytes, CD3, and CD4 reduced, whereas levels of C‐reactive protein, D‐dimer, thrombin time, and lactate dehydrogenase increased in patients with SARS‐CoV‐2 infection. When collecting patient information from multiple studies, because the data were obtained from different hospitals and laboratory platforms have certain differences, different data units were unified and then collated, some indicators have a large difference in normal range, only recorded the abnormal number of people rather than specific values, and others without the specific value also recorded the number of people. The SARS‐CoV‐2 nucleic acid test kit was being developed rapidly, with a clear improvement in sensitivity, based on different biotechnologies. The rapid development and breakthrough of SARS‐CoV‐2 kits were carried out by immediately integrating and collaborating multidisciplinary resources and efforts and by swiftly translating new technologies, gene sequencing, and bioinformatics into clinical practice.^13^ Biochemical phenomes become critical criteria to confirm SARS‐CoV‐2 infection, for example, isolation of SARS‐CoV‐2, real‐time reverse‐transcriptase PCR assay, or SARS‐CoV‐2 full‐genome sequencing.^11^ The latest edition of the National Health Commission, the seventh edition of the new CoV pneumonia diagnosis and treatment program, in addition to nucleic acid detection and sequencing also increased serological testing as the basis for diagnosis. That means detecting new CoV‐specific IgM antibodies and following IgG‐positive or new CoV‐specific IgG antibodies from negative to positive or recovery period from four times higher than the acute phase can also be diagnosed. The genomes of SARS‐CoV‐2 are closely related to those of two bat‐derived SARS‐like CoVs, bat‐SL‐CoVZC45 and bat‐SL‐CoVZXC21, but different from SARS‐CoV (about 79%) and MERS‐CoV (about 50%). However, the homology modeling indicated that SARS‐CoV‐2 has receptor‐binding domain structures, similar to that of SARS‐CoV,^14^ although there are some differences in the key residues.

In addition to diagnosis, clinical phenomes of patients with SARS‐CoV‐2 add significant impact to dynamically differentiate it from other diseases, predict the disease severity, and monitor the therapeutic effect, although the specificity should be furthermore defined. Pulmonary edema with hyaline membrane formation, inflammatory cell infiltration (mainly lymphocytes), multinucleated syncytial cells in alveolar space were observed in lung autopsy tissue of patients with SARS‐CoV‐2 (Table 4), similar to those with SARS‐CoV and MERS‐CoV infections.^16^, ^17^ Liver biopsy specimens from COVID‐19 patients showed moderate microcapsule steatosis and mild lobular and portal activity, which might have been damaged directly by SARS‐CoV‐2 infection or drug‐associated toxicity. Among them, imaging information and genetic detection play important roles. For example, pneumonia of SARS‐CoV‐2 infection should be differentiated from that of mycoplasma pneumonia and community‐acquired pneumonia, which often occur in children and adolescents with a clear seasonal trend. Nucleic acid amplification test can be used in the acute phase of the disease, combined with serological testing, to reduce potential false negative results.^15^ Clinical phenomes of mycoplasma pneumonia mainly include alterations in the lower‐middle lung area, tracheobronchitis, mucinous or mucous purulent sputum, headache, sore throat, rhinitis, and otitis media. The scoring system of clinical phenomes was developed to identify mycoplasma and guide patient management and adjuvant antimicrobial therapy, with 83% sensitivity.^16^ Clinical phenomes of patients with SARS‐CoV included dyspnea, recurrent or persistent fever, new pneumonia infiltration on chest imaging, for example, multifocal airspace merger in the lower lung, and ground‐glass opacity with consolidation.^3^ Patients with MERS‐CoV in 2012 had clinical phenomes including fever, cough, dyspnea, and pneumonia, like any other lower respiratory diseases, but also had rapidly developing acute respiratory distress syndrome, multiple‐organ failure, and death. Compared with SARS‐CoV, MERS‐CoV develops faster into respiratory failure and causes acute kidney damage.^17^

However, there is a lack of clinical phenomes related to the mental health aspect of patients with SARS‐CoV‐2 or SARS‐CoV and MERS‐CoV, suspected people with the infections, as well as the normal population, during the early stage, duration, and late stage of infection. The abnormality of mental phenomes can reflect the living environment and influence the quality of people's lives, although the impact on diagnosis and treatment remain unclear. The COVID‐19‐associated phobia, depression, and over‐responses should be investigated in patients with infection, suspected candidates, relatives, and people in the society. It is important to develop an artificial intelligence evaluation scoring system to dynamically monitor phenomes of mental health as part of the clinical phenomes and enable application to a large number of suspected people before definitive diagnosis and treatment.

Clinical phenomes should be given the priority in the diagnosis and treatment of COVID‐19. The development of SARS‐CoV‐2 infection‐associated phenome list required efficient and professional manpower and a big data‐based technology in a short and high‐pressure period during a sudden outbreak. The artificial intelligence system can help medical staff to automatically collect, achieve, analyze, and optimize databases of clinical phenomes, and offer an immediate response and indication of the patient's health condition. Such an evaluation system will speed up the diagnosis and save a lot of clinical manpower and resources. The digitalized lung imaging analysis system can effectively quantify dynamic changes of the lung tissue and assist clinical judgment and decision‐making. The system should have the capacity for artificial intelligence‐based machine learning to analyze radiomics‐ or imaging‐based data (eg, pathology, computed tomography, ultrasound, and echocardiography), patient‐based information (eg, complaints, feelings, history, and potential factors), clinician‐based records (eg, signs, observations, and questions), and molecular measurements‐based digitals (eg, biochemistry, liquid biopsies, multi‐omics, and RNA and DNA sequencing). The system should also be able to automatically be optimized and updated, to build multi‐dimensional models and perform deep learning, and carry out the application of clinical trans‐omics for diagnosis and treatment of patients with SARS‐CoV‐2 infection. In addition to the role discussed above, artificial intelligence also plays an important role in other aspects. Currently, during the initial implementation, recording travel of people during the outbreak and using the internet and artificial intelligence technology to provide online consultation services based on the information obtained from pre‐examination, artificial intelligence can make a preliminary judgment of the information, and give preliminary advice and recommendations for the doctor's reference. There is also an infrared body temperature monitoring system. Artificial intelligence can automatically analyze images captured by thermal imagers and 3D laser cameras, identify and depict human facial features from the images, measure human forehead temperature, and automatically record it. The driverless delivery vehicle would also be responsible for the distribution of some medical and living materials, which is also inseparable from artificial intelligence technology. Artificial intelligence is also playing an important role in forecasting outbreaks and determining the direction of personnel outflow through data orientation and analysis, as well as assisting drug development. Such a system will be of benefit when aiming to control the outbreak of unknown or unexpected infections in the future.

This article is a preliminary retrospective analysis. When collecting information, the patient information in the raw data was insufficient to allow us to further group the patients. In conclusion, we summarized clinical phenomes of patients with SARS‐CoV‐2 based on published studies to emphasize the importance and specificity of those phenomes in diagnosis and treatment of infection, and evaluate the impact on medical services. Clinical phenomes of SARS‐CoV‐2 infection were compared with those of SARS‐CoV and MERS‐CoV infections. There is an urgent need to develop a system with artificial intelligence‐based machine learning capacity to analyze and integrate radiomics‐ or imaging‐based, patient‐based, clinician‐based, and molecular measurements‐based data, to fight the outbreak of COVID‐19 and enable more efficient responses to unknown infections in the future.

## CONFLICT OF INTERESTS

The authors declare that there is no conflict of interests regarding the publication of this paper.

## Supporting information

Supporting Information.
